# Consumer Insights to Eco-Design a Hot Sauce: Understanding Household Use for Product Optimization through Focus Groups and a Home-Use-Test Study

**DOI:** 10.3390/foods13060945

**Published:** 2024-03-20

**Authors:** Paula Torán-Pereg, Elena Romeo-Arroyo, Stéfani Novoa, Guillermo Pardo, Laura Vázquez-Araújo

**Affiliations:** 1 BCC Innovation, Technology Center in Gastronomy, Basque Culinary Center, 20009 Donostia-San Sebastián, Spain; ptoran@bculinary.com (P.T.-P.); eromeo@bculinary.com (E.R.-A.); snovoa@bculinary.com (S.N.); 2 Basque Culinary Center, Faculty of Gastronomic Sciences, Mondragón Unibertsitatea, 20009 Donostia-San Sebastián, Spain; 3 Basque Centre for Climate Change (BC3), 48940 Leioa, Spain

**Keywords:** food development, sustainability, home-use-test, hot sauce, qualitative research, consumer-centric

## Abstract

Promoting sustainable choices requires making food with proper environmental performance readily available to consumers, but these products must be appealing to ensure market success. The aim of the present study was to investigate the acceptability and perception of an eco-designed product using a home-use-test approach to identify its desired extrinsic features and to better understand how consumers would use the product in a real consumption context. First, three focus groups were conducted to identify the key perceived aspects of the product. A consumer survey was designed with the information gathered from the focus groups, and a home-use-test was then performed (*n* = 207). Results showed high acceptance of the product, as well as its potential corrections, e.g., to thicken the texture of the sauce. A total of 64% of consumers expressed their willingness to switch from a well-known brand to the new developed product, and this hypothetical shift could lead up to a 58% reduction in greenhouse gas emissions associated with the product use. Understanding consumer expectations at every design stage helps the development of market-viable and sustainable products, and the present research proposes an interesting methodology that can be effectively applied during the final stages of eco-designed food development.

## 1. Introduction

Due to the environmental impact exerted by the food system, stakeholders from the food value chain are called to adopt more sustainable practices [1,2]. From the perspective of household consumption, food (48%) has been identified as the most impactful area in European households, surpassing housing (19%), mobility (15%), household goods (11%), and appliances (6%) [3]. The food industry is investing significant efforts in developing new food products made under more sustainable approaches (e.g., organic raw materials, new plant-based products, increasing the use of alternative protein). However, consumer selection of sustainable foods remains limited, primarily driven by people with strong environmental concerns and motivations [4,5,6]. Some authors have suggested that societal barriers (e.g., lack of interest and awareness, skepticism, or pricing issues) pose greater challenges than technical barriers in reaching the desired circular economy [7,8]. A larger number of consumers who engage with sustainable choices are necessary to boost the transition to a more sustainable food system. Making eco-designed foods readily available can simplify this transition, even for consumers who are not fully aware of their positive impact.

Food choices are shaped by a combination of product, individual, and societal factors [9]. Consumers’ expectations and perceptions are directly influenced by the intrinsic (e.g., flavor, aroma, texture, nutritional quality) and extrinsic properties (e.g., labels, claims, packaging attributes) of the products. Several studies have shown that the intrinsic sensory properties of foods are often primary drivers of acceptance [10,11]. However, the impact of packaging design on product perception is also significant. Different authors have suggested that the appearance [12], the tactile properties of packaging, or the messages linked to the product [13] may significantly affect food choice and perception. These insights underscore the importance of addressing both intrinsic and extrinsic factors in the design of food products to meet consumer demands and ensure market success.

Food design is a discipline that aims to create consumer-desirable foods. Traditionally driven by designers, during the last years, there has been a growing recognition of the benefits of interdisciplinary collaboration (involving, e.g., designers, scientists, and consumers) to address challenges related to food consumption and to enhance the impact of the designed foods in society [14,15]. Eco-design principles have been suggested to be included into three main categories, depending on the food chain stage: “design for sustainable sourcing” (e.g., selecting a specific cultivar), “design for optimized resource use” (e.g., selecting a raw material or energy source for production), and “design for end-of-life optimization” (linked to reuse/recycling) [16], and consumer behavior may have an important impact specially on the first and the third ones. Therefore, interdisciplinary food design is key in promoting sustainable food choices. While the value of interdisciplinary approaches has been acknowledged, the existing literature lacks in-depth case studies showing detail on the different methods that could be used to gather consumers’ opinions about the different features that contribute to optimizing a product from a sustainability perspective.

The aim of the present research was to assess the acceptability and gather information about the home-use of a product that had previously been designed using a consumer-centric approach [17,18]. From the product conceptualization and design phases, a new food was developed: a hot sauce made from unexploited green peppers. Insights from the food value chain stakeholders were used to choose the raw materials (“design for sustainable sourcing” eco-design principle) [17], and environmental assessment tools (Life Cycle Assessment) were used to discard some prototypes and select the final one [18]. This approach allowed for improving the intrinsic characteristics of the product from a sustainability point of view, without compromising its sensory properties. The present study contributes to the final food design stages (linked to the use, “reuse/recycling” eco-design principle), showing a consumer-centered approach to measure acceptance and identify the potential communication strategies to promote the selection of an eco-designed product.

## 2. Materials and Methods

The protocol for the consumer study was approved by the ethics committee of Mondragon Unibertsitatea (IEB-20221115). All articles from the Declaration of Helsinki and the 2016/679 EU Regulation on the protection of natural persons regarding the processing of personal data and on the free movement of such data were met. Each participant was provided an explanation of the experimental process, and informed consent was obtained from all subjects involved in the study.

### 2.1. Insights from Consumers and Chefs for Questionnaire Design: Focus Groups

Focus groups have been recognized as a useful phase prior to launching a consumer study [19]. Thus, three 90 min focus groups were conducted with the objectives of (i) understanding the perception of the designed product (green pepper hot sauce) from a qualitative standpoint, (ii) identifying the potential uses/applications of the product, (iii) identifying some key extrinsic features (e.g., packaging properties, communication messages). Participants were recruited by publishing an advertisement on social media; the recruitment criteria were to be an adult and a regular consumer of hot sauces (at least once per week). A total of 26 people (15 consumers and 9 gastronomy professionals) participated in the focus groups, divided into two sessions with regular consumers and a third session with professionals from the gastronomy sector. Besides this professional background, groups were heterogeneous, and people from different age groups, genders, and professions were represented in the sessions (from 23 to 51 years old; 62% males and 34% females; from 4 to 20 years of experience in the gastronomy sector for the professionals’ group). Consumers were not grouped with gastronomy professionals to prevent the possibility of bias in their opinions. The sessions were guided by two trained moderators and were conducted in April–May 2023. The number of participants and conducted focus groups was decided following literature recommendations: from 3 to 12 participants per focus group and conducting focus groups until redundant information was collected [19].

**Figure 1:**
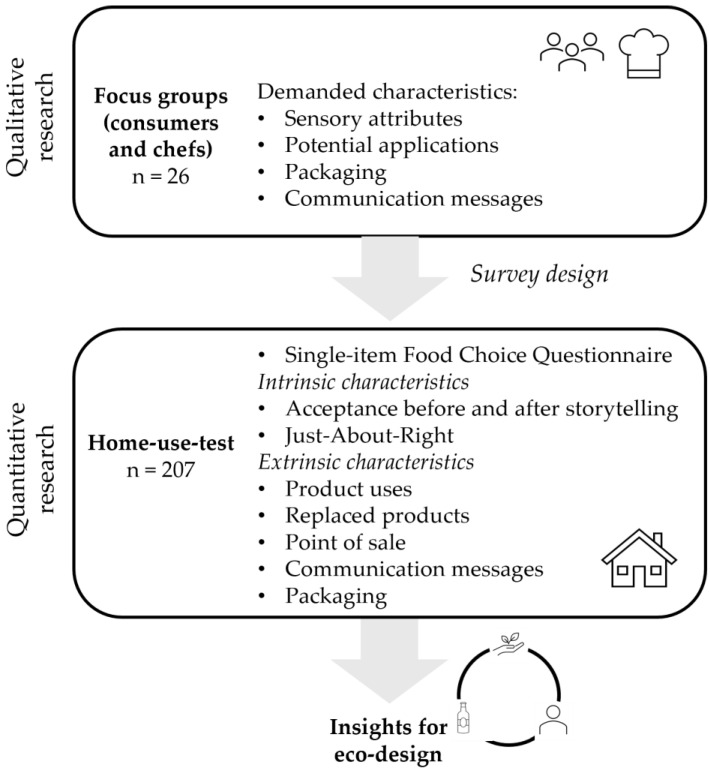
Scheme of the procedure followed in the present research.

Each session started by contextualizing the environmental impact of the food system and presenting the developed product. Then, some general aspects of the “sauces” food category were discussed. Subsequently, the discussion was guided through four thematic blocks with different interventions: (i) sensory attributes, (ii) culinary applications, (iii) packaging, and (iv) product communication. A sample of the developed product, as well as other commercial hot sauces, were available during the session for participants to taste. The sessions lasted 1.5 h and were audio-recorded, transcribed, and analyzed by three researchers. The collected information was used to design the questionnaire for the subsequent consumer study (Figure 1).

### 2.2. Consumer Study: Home-Use-Test (HUT)

To allow potential consumers to try the product in a real consumption context and explore its uses, a home-use-test was conducted. Participants were recruited from BCC Innovation’s consumer database. Consumers received a sample of the sauce (40 mL, transparent glass jars) with instructions to follow the test and labeled with a QR code that gave access to the questionnaire. The RedJade^®^ software v.5.1.1 (RedJade Sensory Solutions, LLC, Palo Alto, CA, USA) was used to collect responses.

A total of 207 responses were collected (54% women, 45% men, 1% non-binary; 25% from the 18–30 years old group, 40% from the 31–45 y/o group, 25% from the 46–60 y/o group, and 10% over 61 y/o; 54% from Euskal Herria, 46% from other locations). The first part of the questionnaire included demographic questions on gender, age, location in which they had been living during the last 5 years, profession, and the single-item Food Choice Questionnaire [20], in which participants were required to assess their agreement/disagreement with 11 different food choice motivations on a 7-points scale (where “1 = completely disagree”, “4 = neither agree, nor disagree”, and “7 = completely agree”). Then, participants were requested to test the product “seasoning the food they wanted, and at the time they considered appropriate for that product” and to answer the following questions, which were proposed after analyzing the results of the focus group phase:

(a) To rate acceptance, flavor, color, and consistency on a 9-points hedonic scale (1 = dislike extremely, 5 = neither like nor dislike, 9 = like extremely)
(b) Just-About-Right (JAR) questions on spiciness, sweetness, sourness, smoke aroma, and consistency (1 = too low, 4 = just about right, 7 = too much).
(c) Then, the storytelling of the product design was presented: “*the sauce you have tried contains green pepper discarded during the production of Espelette Protected Designation of Origin pepper as main ingredient. These peppers were discarded for not having 80% of the skin surface in red color. Due to its green color, it could not be labeled as “Espelette pepper D.O.P.”, causing a lack of demand or a commercial outlet for this raw material. It is estimated that 20% of pepper production is discarded for this reason, therefore, the product you have just tried, made with this raw material, can contribute to reducing food loss. During the development of the product, the environmental impact derived from its production (ingredients, processes, packaging, etc.) was considered. After analyzing different formulas, we have reduced the environmental impact of the product by 54–91%, when compared to the initial formula.*”, and participants were asked to rate the product acceptance again (9-points hedonic scale).
(d) Three Check-All-That-Apply (CATA) questions to determine: (i) the products that could potentially be replaced by the developed sauce, (ii) the culinary applications of the product, and (iii) preferred points of sale to purchase the product.
(e) To indicate the information that would motivate the purchase of the sauce if reflected on the packaging (7 points scale, where “1 = I would not be motivated at all” and “7 = I would be very motivated”). The list of motives was obtained from the focus group phase and is shown in the corresponding results paragraph.
(f) Finally, participants were requested to choose between two types of packaging (glass vs. plastic) and to specify the reason for their selection using a CATA question.

### 2.3. Data Analysis

Hierarchical Cluster Analysis, using Euclidean distance and Ward’s criterion of aggregation was conducted with the single-item Food Choice Questionnaire (FC) to determine the groups of consumers that shared a common FC profile. One-way analysis of variance (ANOVA) followed by a post hoc test (Tukey’s HSD) was conducted to determine differences in the food choice motivations (section a of the questionnaire), messages that drew different interest among consumers (section e of the questionnaire), and all responses among clusters of consumers and other demographic segments when the dataset allowed the analysis (gender, age, location). All statistical analyses were conducted using XLSTAT Version 2023.3.0 (Addinsoft, Paris, France) [21]. Differences were considered significant when *p* < 0.05 unless otherwise stated in the results section.

## 3. Results and Discussion

### 3.1. Focus Group Results

Focus group results allowed for a better understanding of consumers’ perception of the product. Although separate focus groups were conducted for gastronomy professionals and consumers, their responses largely aligned across all sections except for “potential applications”. Therefore, the results are presented in a single table, segmented by section for clarity (Table 1). In general, the product was described as “innovative” and different from other products from the same category. During the first section (sensory attributes), participants identified the dominant organoleptic attributes of the product and the category, such as spiciness, red-purple color, thin consistency, fruity and smoky flavor, and sweetness, expressing varying levels of liking. When asking participants about attribute expectations on a “sustainable hot sauce”, different intrinsic and extrinsic attributes were mentioned. A “weak flavor profile” and “mild hot intensity” were expected in a sauce promoted as “sustainable” versus a conventional one; “natural flavor”, “freshness” or “simplicity of ingredients” were also expected in this kind of product. During the second section of the focus group (potential applications), participants mentioned diverse uses, from the fast-food and snacking category (e.g., pizza, hamburgers, nachos) to refined meals (fish, cheese, game recipes). The chefs and gastronomes group mentioned a wider range of culinary applications, providing some examples of cooking processes and recipes that could be used to communicate the potential of the product. The third section (packaging) elicited less discussion, with a significant consensus among participants on the best packaging properties: small-sized, transparent, and made of glass, because glass was perceived to be of “higher quality and more sustainable”. In the fourth section (product communication), participants expressed interest in highlighting the chili cultivar chosen for the product and the level of spiciness. Likewise, the origin of the product was considered important, as a “local” product could be perceived as more sustainable because it supports the local community, an extrinsic value that may outweigh other environmental concerns. Participants remarked that excessive use of the word “sustainable” or using certain claims or labels could be perceived as “greenwashing”. Finally, participants noted that “sustainable products” could be perceived as more expensive or of lower quality than products of the same category without this claim and remarked that, in general, the organoleptic characteristics would play a more important role than the sustainability-related claims.

From all these inputs, the questionnaire for HUT was developed, including the following: the list of different sensory properties mentioned by respondents of the focus groups (sections a and b of the questionnaire); a storytelling of the product (section c); CATA questions to identify applications/uses of the product that included the concepts mentioned during the focus groups (section d); a list of potential information concepts that could motivate the product purchase, including the sentences “information on using raw materials that were going to be discarded (green peppers) and the avoidance of this waste thanks to the sauce production”, “the detailed list of ingredients: peppers, cranberries, etc.”, “information on the organoleptic profile of the product (e.g., flavor, level of pungency, smoky aroma, etc.)”, “recommendations for use (e.g.,: recipes)”, “information about the origin of the product (e.g.,: Espelette, Euskal Herria)” and “the environmental footprint of the product” (section e); and a question to assess two kinds of packaging.

### 3.2. Consumer Study: Home-Use-Test

The Hierarchical Cluster Analysis showed three clusters of consumers from the Food Choice questionnaire results; Cluster 1 (*n* = 96) was characterized by scoring all FC items with higher scores than Cluster 2 (*n* = 75) and Cluster 3 (*n* = 36). Table 2 shows significant differences found in the response of the single-item Food Choice Questionnaire by cluster and among clusters; these data provided information on the main food choice drivers of the consumer segment participating in the present study. The differences among clusters should be considered tentative, because of the different *n* of the clusters. In general, the main FC drivers, “provides me with pleasure sensations” and “healthy”, were common for all clusters, and results agreed with the Eurobarometer survey results, in which “taste” was the main driver (45% of responses). The Eurobarometer survey showed that food safety (42%) and cost (40%), were also identified as important food purchase motivations among European consumers [22]. The least important FC motive for the different clusters was “a way of monitoring my mood”, confirming previous studies in which the Spanish population was not characterized by having a great % of “emotional eaters” [23,24]. C3 was clearly different from C1 and C2 because of the lower scores given to the “Environmentally friendly” and “Fairly traded” items, items which could be directly linked to a “sustainable” product. “Affordable” and “Convenient” were considered more important for C3 that for C1 and C2.

Table 3 shows the results of sections *a* and *b* of the questionnaire (acceptance and JAR questions). Statistical analyses showed no significant differences among FC clusters or other demographic segments (gender, age, location) for the hedonic response and the JAR questions. In general, results suggested that the product was liked, all attributes being over the “neither like nor dislike” score. These results were promising, especially considering that taste is often perceived as a trade-off in sustainable food options [25]. JAR results indicated that for most consumers (50–71%), the studied attributes (spiciness, sweetness, acidity, smoky aroma, and consistency) were perceived as “Just About Right”. The attribute with the higher improvement opportunity was consistency, because 42% of participants perceived the product as too thin; in addition, 22% and 24% of respondents perceived the sauce as too spicy and the smoky aroma too high, respectively, suggesting other potential areas of improvement. Liking mean drops for the proportion of consumers who rated the sauce as too spicy, too intensely smoky, and too thin were below 0.6 in all cases, suggesting that these considerations may not affect acceptance of the product. Finally, knowing about the product-making process characteristics (storytelling) had a positive impact on product acceptance, because liking significantly (*p* < 0.05) increased from 6.11 to 6.52, as well as the % of respondents scoring overall liking over 7 (from 49 to 62%). The impact of different messages and logos on food perception and choice has been widely studied. Yang et al. [25] investigated the impact of extrinsic information on snacks made with Bambara, an African legume known for its low environmental impact. Although no significant differences in liking were observed when the product was tested with/without information about its sustainable features, significant differences were shown in the emotional responses elicited by the product. Consumers reported experiencing more positive emotions when informed about the environmental benefits of the product, suggesting that such knowledge could influence product purchase. Previous studies have shown that specific consumer segments gave higher ratings to apples labeled as “organic” or “local” than to those not labeled, although the samples were the same [26]. On the other hand, another research reported no effect on using sustainability or healthy claims on liking scores of spreads made from discarded orange peels, indicating that the sensory properties were the major driver in the case of this specific product [13]. These contradictory results found in the literature suggest that sustainability claims could be important depending on the product category or maybe the targeted consumer segment.

Responses to the CATA questions on product application, potential substituted product, and purchase location (section d of the questionnaire) showed that the main application would be to use it for seasoning meats, that 64% of consumers considered the new product a potential substitute for Tabasco^®^ sauce, and that the preferred location to purchase the product would be a supermarket (Table 4). Sauces sales in Spain summed to a total of 322,641 tons of sauce in 2019, of which 0.7% were hot sauces [27]. No specific data have been found on Tabasco^®^ sales, but considering its extended popularity, to calculate the carbon footprint, it has been assumed that 50% of the hot sauce sold in Spain could be Tabasco^®^, representing total sales of 1131 tons. The CarbonCloud database estimates a carbon footprint of 2.91 kg CO_2_-eq kg^−1^ for Tabasco pepper sauce considering the agriculture and processing phases (transport and packaging excluded) [28]. The sauce developed during the present research had a carbon footprint of 0.47 kg CO_2_-eq kg^−1^ when considering the same production phases [18]. The comparison between the products’ impacts should be cautiously considered due to methodological differences in these environmental impact calculations, the sales estimations, and production capacity of the new product, but the reduction in emissions could be estimated from 3287 Tn CO_2_-eq to 1527 Tn CO_2_-eq (a 54% reduction), if 64% of Tabasco consumers shifted their purchasing choice to the newly developed product.

Most of the respondents stated that they would consume the sauce with meat (77%), pizza (39%), and vegetables (38%). Different studies have reported that adding sauces and seasoning could increase food intake in senior adults, who sometimes suffer from lack of appetite [29] and that familiar seasoning such as ketchup could increase acceptance of novel foods in children [30]. No significant differences were found in the responses given by the different age or other demographic segments, although further analysis could be conducted to determine eating behavior in specific population groups when incorporating this kind of product in the recipe. Finally, consumers chose “supermarkets” (80%), “gourmet stores” (50%), and “specialty stores” (44%), as their preferred points of sale for the new product.

**Figure 2:**
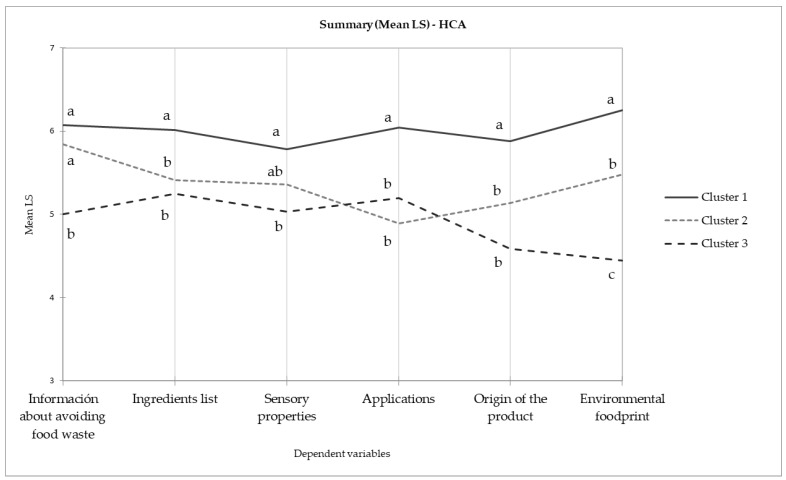
Messages that could motivate product selection by FC clusters (section e of the questionnaire). Different letters in the same item show significant differences (*p* < 0.05) among clusters (Tukey’s HSD).

In general, no significant differences were found among information items that would motivate consumers to purchase the product if shown in the packaging (section e of the questionnaire), but significant differences were found among FC clusters (Figure 2). Consumers from C1 showed high interest for all the presented concepts; consumers from C2 showed less interest on the “ingredients list”, “applications”, “origin of the product”, and “environmental footprint”-related information than C1. Finally, C3 showed a similar behavior than C2, but showed less interest in the messages linked to the “environmental footprint” and information about “avoiding food waste generation” than the other two clusters. These results should be considered tentative because of the different sample size of the clusters, but further research should be conducted to explore the best way of sensibilizing C3 individuals and to promote sustainable food choices among this population segment because it represented 17% of the participants. These findings underline the importance of tailored communication strategies in marketing and of aligning messages with the distinct interests of each consumer cluster, which may lead to more engagement and a higher impact on sustainable foods selection. Different studies have shown that selecting local food could be motivated by consumers perceiving it as having superior sensory quality [31,32,33] or because they have a strong emotional link with the region [34], instead of having environmental-related concerns. Although 85% of European consumers would appreciate a logo to easily recognize sustainable and healthy foods [22], some studies on consumers’ perception of carbon footprint showed limited understanding of its significance and a limited impact on consumers’ choices [35,36], so the logo information should be cautiously considered. Other kinds of strategies, such as decreasing prices and taxes, have been suggested as effective strategies for shifting food choices to more sustainable options [37].

Finally, the last question of the survey (section f) revealed that most of the participants preferred glass (96%) over plastic packaging (4%), with the most frequently cited reasons being, “because it is more sustainable” (64%), “because it is of higher quality” (62%), “because it is easier to recycle/dispose” (56%) (Table 5). These findings agreed with other studies on consumers’ perception of different packaging materials; glass has been reported to be the most valued material due to its appearance and “ecological” perception [38], although some authors have suggested that this preference could be influenced by the extended knowledge about the high recyclability rate of glass among citizens [39]. However, glass recyclability has some drawbacks due to the high energy requirements for the glass melting process and subsequent production of new containers [40], although it is possible that consumers are not completely aware of this information. In addition, deficiencies in management and disposal of single-use plastic packaging have resulted in negative impacts on the environment, wildlife, and human health [41,42], increasing consumers’ concern about this kind of material. In addition, its sourcing from fossil fuel presents an important challenge in relation to sustainability. Different studies have reported that consumers link plastic packaging with low-quality products and, therefore, have a negative perception of this kind of material [43,44], but plastic packaging could have a better performance than glass depending on the specific case study [45,46]. All these highlight the need for effective communication strategies regarding the selection of the best packaging material to promote the most sustainable option for each specific product.

The different data collected during the present research were useful to better understand consumer perception of the developed product and provided useful insights to finish the design and development process. Some aspects were not addressed, such as “willingness to pay”, but the approach used in the present research exemplifies the potential of consumer research for food eco-design. Using a HUT approach to explore the consumption of the product in a real context allowed us to collect data about the products that were consumed with the developed sauce, providing additional information which could be used to expand the study of the environmental impact of the developed product. Further research should be conducted to measure the current impact of the selected packaging vs. the most sustainable but attractive options, as well as consumers’ behavior regarding packaging disposal and/or recycling. Also, new product categories should be explored, because different foods might need specific methodologies to explore its applications and household use. Finally, a comparative analysis of various food design methodologies could equip food companies and designers with valuable insights, enabling them to select the most appropriate approach based on available time and resources.

## 4. Conclusions

Conducting focus groups in which the sauce prototype was presented allowed consumers to identify key aspects of the new developed product, facilitating the subsequent design of a consumer survey. The data collected using home-use-test (HUT) indicated a general acceptance of the new product, suggesting its potential market viability but also the potential areas of improvement. The HUT approach allowed consumers to try the product with different foods, allowing the collection of real-context feedback. The developed product could be a potential substitute for a well-established sauce in the market, and this substitution could result in a significant reduction of greenhouse gas emissions. This kind of approach could be used in other food design processes to promote sustainable products’ development and consumption. In addition, the assessment of different methodologies for success in the eco-design and development of foods should be further investigated. The use of qualitative (focus groups) and home-use quantitative consumer research has been showcased in the present study; however, further research is needed to explore the suitability of diverse methodologies across different product categories. Ideally, this would establish best practices for gathering consumer data for optimal consumer-centric eco-design approaches.

## Figures and Tables

**Table 1:** Summary of the qualitative information obtained during the focus groups.

Intervention	Concepts
Sensory descriptors/attributes	Red-purple color, thin consistency, fruity and smoky flavor, sweetness. Expectations on a “sustainable sauce”: natural flavor, freshness, weak flavor profile, slightly hot, short ingredients list.
Potential applications	Nachos, pizza, burger, fish, meats, poultry, poke bowl, pasta, rice, gyozas, tacos, mussels, vinaigrettes, marinades, vegetables (eggplant) cheese, potatoes, mixed with other sauces, etc.
Packaging	Small volume. Transparent, to show the color of the product. Mainly glass; optional, some carboard (because of providing a higher sustainable/quality impression).
Product communication	Origin or the raw materials and manufacturing process; support to local producers. Ingredients: fruit content, type of chili. Avoid overuse of environmental claims/labels because it might cause rejection, distrust, and consumers believing that the product is more expensive.

**Table 2:** One-way ANOVA results of the Food Choice Questionnaire. Different letters indicate different post hoc groupings by Tukey’s HSD, lowercase to show differences among clusters (row) and uppercase to indicate significant differences within each cluster (column) (*p* < 0.001).

FCQ Item	C1	C2	C3
Healthy	6.77 a/A	6.03 a/AB	5.11 b/AB
Fairly traded	6.70 a/AB	5.60 b/B	3.30 c/CD
Provides me with pleasurable sensations	6.69 a/AB	6.43 ab/A	5.64 b/A
Natural	6.65 a/AB	5.67 b/B	4.78 c/ABC
Environmentally friendly	6.63 a/AB	5.55 b/B	3.67 c/D
Animal friendly	6.40 a/ABC	4.90 b/CD	3.55 b/D
Affordable	6.30 a/BC	5.41 b/BC	5.30 b/AB
Convenient (in buying and preparing)	6.01 a/C	5.53 ab/BC	4.89 b/AB
Helps me control my weight	6.00 a/C	4.39 b/D	4.50 b/BCD
Familiar	5.46 a/D	4.44 b/D	4.28 b/BCD
A way of monitoring my mood	5.40 a/D	4.50 b/D	3.67 c/D

C1 = Cluster, C2= Cluster 2, C3 = Cluster 3.

**Table 3:** Results of acceptance of the intrinsic sensory attributes of the sauce, sections *a* and *b* of the HUT questionnaire.

Acceptance	Mean Score ± SD	% Consumers		
		Do Not Like (Scores 1–3)	Neither Like nor Dislike (Scores 4–6)	Like (Scores 7–9)
Overall liking (OV)	6.11 ± 1.77	9	42	49
Flavor	5.97 ± 1.90	12	41	47
Color	6.70 ± 1.97	8	29	63
Consistency	5.77 ± 2.02	16	40	44
OV after storytelling	6.52 ± 1.93	10	28	62
Just-About-Right		Low (scores 1–3)	JAR (scores 4–6)	High (scores 7–9)
Spiciness	5.10 ± 1.77	16	62	22
Sweetness	4.79 ± 1.61	17	71	12
Sourness	5.35 ± 1.51	10	69	21
Smoky aroma	5.08 ± 1.93	21	55	24
Consistency	4.06 ± 1.71	41	50	9

**Table 4:** Percentage of consumers marking the different items of the CATA questions on product application, potential substituted products, and purchase location (section *d* of the questionnaire).

Substituted Product	%	Application/Use	%	Point of Sale	%
Tabasco^®^	64	Meat	77	Supermarket	80
BBQ sauce	37	Pizza	39	Gourmet store	50
Ketchup	27	Vegetables	38	Specialty store	44
Sriracha	18	Rice	29	Restaurant/Bar	36
Mustard	14	Pasta	27	Market	28
Kimchi	11	Fish	19	Others	4
Valentina	11	Seafood	14		
Mayonnaise	7	Dairy	6		
Soja	5	Fruits	5		
Gochujang	4	Others	11		
Others	4				
None	14				

**Table 5:** Reasons for choosing the selected packaging (glass, 96%; plastic, 4%).

Reason	% of Respondents
It is more sustainable	64
Gives more “quality” image	62
It is easier to recycle/dispose	56
The product is better preserved in this packaging	54
It is prettier	48
It is more typical in this product category	47
It is easier to use	39
Requires less space	12
Weighs less	4
It is cheaper	2
Other	1

## Data Availability

The original contributions presented in the study are included in the article, further inquiries can be directed to the corresponding author.
